# A new mouse mutant with cleavage-resistant versican and isoform-specific versican mutants demonstrate that proteolysis at the Glu^441^-Ala^442^ peptide bond in the V1 isoform is essential for interdigital web regression

**DOI:** 10.1016/j.mbplus.2021.100064

**Published:** 2021-05-14

**Authors:** Sumeda Nandadasa, Cyril Burin des Roziers, Christopher Koch, Karin Tran-Lundmark, María T. Dours-Zimmermann, Dieter R. Zimmermann, Sophie Valleix, Suneel S. Apte

**Affiliations:** a Department of Biomedical Engineering-ND20, Cleveland Clinic Lerner Research Institute, 9500 Euclid Avenue, Cleveland, OH 44195, United States; b Institut Cochin, Inserm U1016 - CNRS UMR8104 - Paris Descartes University Medical School, 24, Rue du faubourg Saint Jacques, 75014 Paris, France; c Department of Experimental Medical Science and Wallenberg Center for Molecular Medicine, Lund University, Lund, Sweden; d Department of Pathology and Molecular Pathology, University Hospital Zurich, Zurich, Switzerland

**Keywords:** Extracellular matrix, Metalloprotease, Proteoglycan, ADAMTS, Limb development, Syndactyly

## Abstract

Two inherent challenges in the mechanistic interpretation of protease-deficient phenotypes are defining the specific substrate cleavages whose reduction generates the phenotypes and determining whether the phenotypes result from loss of substrate function, substrate accumulation, or loss of a function(s) embodied in the substrate fragments. Hence, recapitulation of a protease-deficient phenotype by a cleavage-resistant substrate would stringently validate the importance of a proteolytic event and clarify the underlying mechanisms. Versican is a large proteoglycan required for development of the circulatory system and proper limb development, and is cleaved by ADAMTS proteases at the Glu^441^-Ala^442^ peptide bond located in its alternatively spliced GAGβ domain. Specific ADAMTS protease mutants have impaired interdigit web regression leading to soft tissue syndactyly that is associated with reduced versican proteolysis. Versikine, the N-terminal proteolytic fragment generated by this cleavage, restores interdigit apoptosis in ADAMTS mutant webs. Here, we report a new mouse transgene, *Vcan*^AA^, with validated mutations in the GAGβ domain that specifically abolish this proteolytic event. *Vcan*^AA/AA^ mice have partially penetrant hindlimb soft tissue syndactyly. However, *Adamts20* inactivation in *Vcan*^AA/AA^ mice leads to fully penetrant, more severe syndactyly affecting all limbs, suggesting that ADAMTS20 cleavage of versican at other sites or of other substrates is an additional requirement for web regression. Indeed, immunostaining with a neoepitope antibody against a cleavage site in the versican GAGα domain demonstrated reduced staining in the absence of ADAMTS20. Significantly, mice with deletion of *Vcan* exon 8, encoding the GAGβ domain, consistently developed soft tissue syndactyly, whereas mice unable to include exon 7, encoding the GAGα domain in Vcan transcripts, consistently had fully separated digits. These findings suggest that versican is cleaved within each GAG-bearing domain during web regression, and affirms that proteolysis in the GAGβ domain, via generation of versikine, has an essential role in interdigital web regression.

## Highlights

• • A novel *Vcan* mouse allele, *Vcan*^AA^, has ADAMTS protease-resistant versican.
• • *Vcan*^AA/AA^ mice are viable and develop soft tissue-syndactyly (STS)
• • *Vcan*^AA/AA^ STS is rendered more severe in combination with *Adamts20*^Bt/Bt^.
• • Mice lacking the versican GAGβ domain, but not the GAGα domain, also have STS.
• • The versican GAGβ proteolytic fragment versikine is necessary for web regression.

## Introduction

Extracellular matrix (ECM) proteolysis is necessary for tissue remodeling during mammalian development. For example, branching morphogenesis, cardiac valve leaflet formation, endochondral ossification and interdigital web regression each require precisely timed and spatially controlled tissue remodeling, which was demonstrated by impairment of these processes in mice with engineered mutations in specific proteases [1], [2], [3], [4], [5], [6]. A number of secreted and cell-surface metalloproteases are implicated in ECM remodeling [7]. Because protease function is realized through substrate cleavage, identifying the most impactful cleavages will elucidate potentially important biological pathways. However, since few proteases have a single substrate, an inherent challenge in interpreting protease-deficient phenotypes is determining which of many possible reduced proteolytic cleavages are responsible for an observed phenotype. A corollary to this statement is that the phenotype may result from reduced proteolysis of several substrates, not just a single one, requiring stringent analysis of each substrate to determine the precise contribution of each. The most rigorous validation, indeed a proof, of the importance of cleavage of a substrate would be partial or complete recapitulation of a phenotype observed in a protease-deficient mutant by a cleavage-resistant substrate [8]. However, such experiments are infrequently undertaken because of the high level of risk involved, since many proteases may attack a protein and a protease could cleave the protein at many sites. This risk reduces the likelihood of definitive insights that could be obtained from rendering a single site resistant to proteolysis. Therefore, defining the significance of specific cleavages in substrates has generally not reached the highest levels of experimental rigor. As a result, the functional landscape of proteolysis during embryonic development, physiological processes and diseases is incompletely annotated.

Versican is a large, aggregating chondroitin sulfate (CS) proteoglycan which is a major constituent of ECM, especially provisional ECM [9]. It is essential for survival beyond 10 days of gestation in the mouse and is required specifically for early development of the entire circulatory system, i.e., the heart, blood vessels and blood cells [10], [11]. It forms networks in ECM via two main mechanisms, i.e., by binding hyaluronan through its N-terminal G1-domain and by interacting with numerous ECM components including fibronectin, fibrillins, fibulin-1 and −2 and tenascin-R and -W via its C-terminal G3 domain (reviewed in [12], [13]).CS chains are covalently attached to large, alternatively spliced core protein domains between the G1 and G3 domains, named GAGα (encoded by *Vcan* exon 7) and GAGβ (encoded by *Vcan* exon 8) [14]. Alternative splicing of these exons generates distinct versican variants: V0 (containing both GAGα and GAGβ domains), V1 (containing only the GAGβ domain), V2 (containing only the GAGα domain) and V3 (containing neither GAG-bearing domain). Because of its abundance in embryonic tissues and crucial role in embryogenesis, proteolytic turnover of versican has elicited considerable interest [12].

Among their diverse substrates identified in a variety of biological settings [2], [15], several ADAMTS proteases cleave versican at specific peptide bonds, i.e., E^441^-A^442^ (in the GAGβ domain, human and mouse versican V1 isoform enumeration) and E^405^-A^406^ (in the GAGα domain, human and mouse versican V2/V0 isoform enumeration) [16]. After proteolysis at E^441^-A^442^, the resulting new C-terminus, DPEAAE^441^ (DPEAAE^1428^ in the V0 isoform), is specifically detected by a neoepitope antibody, anti-DPEAAE [17], which is widely used to demonstrate versican proteolysis in situ, since this epitope is conserved in humans and mice. The corresponding GAGα domain cleavage in human versican could be recognized by another neo-epitope antibody, anti-NIVSFE [16], whose immunogenic sequence is not conserved in the mouse. ADAMTS 1,4,5,9,15 and 20 can digest versican at the GAGβ site [18], [19], [20], [21], [22] and mouse mutants lacking these proteases demonstrated a requirement for versican GAGβ processing during diverse developmental processes (reviewed in [12]). These processes include interdigital web regression (ADAMTS5, ADAMTS9 and ADAMTS20) [4], [23], myocardial compaction (ADAMTS1) [24], cardiac valve sculpting (ADAMTS5) [25], midline facial closure (ADAMTS9 and ADAMTS20) [23], [26], [27], [28], neural tube closure (ADAMTS9 and ADAMTS20) [28], melanoblast colonization of hair follicles (ADAMTS9 and ADAMTS20) [20], [27], [29], skeletal myotube formation (ADAMTS15) [22], vascular development (ADAMTS1, ADAMTS5 and ADAMTS9) [27], [30], [31] and myometrial activation prior to parturition (ADAMTS9) [32]. The N-terminal G1 domain-bearing versican V1 fragment that extends to the GAGβ cleavage site is referred to as versikine [4], whereas the corresponding versican V2 fragment was previously described as glial-hyaluronic acid-binding protein (GHAP) [16]. In contrast to GAGβ processing, GAGα proteolysis has been less extensively studied and no specific function is yet ascribed to GHAP.

The evidence linking ADAMTS proteases to versican processing in interdigit webs, and the importance of versican proteolysis in this context is compelling. *Adamts5, Adamts9* and *Adamts20* mRNAs are co-expressed at heightened levels in interdigital web mesenchyme just prior to web regression [4]. Moreover, reduced anti-DPEAAE staining of interdigital webs during the period during which regression occurs was evident in combined *Adamts5 + Adamts20* mutant mice, which consistently develop soft-tissue syndactyly (STS) and in mice with limb-specific *Adamts9* deletion [4], [23]. Impaired interdigital web regression in these mutants was associated with persistence of versican-rich ECM and a subsequent failure of apoptosis [4], [23]. The N-terminal G1 domain-containing fragment (versikine), was found to induce cell death during interdigital web regression in *Adamts5 + Adamts20* mutants, and deletion of one *Vcan* allele in *Adamts20*^bt/bt^ mice led to more severe STS, suggesting that versican itself, albeit after proteolysis, could be essential for web regression [4]. Taken together, the findings suggest that cooperative versican processing by ADAMTS proteases generates a critical level of versikine to promote apoptosis of interdigital mesenchymal cells; conversely, when versican proteolysis is reduced below this hypothetical critical threshold, interdigit cells may be resistant to BMP-induced apoptosis [4].

Here, we report the generation and analysis of mice with versican resistant to cleavage at the E^441^-A^442^ ADAMTS site, using a technically different approach than that recently utilized to generate another cleavage-resistant *Vcan* allele (designated *Vcan*(R/R) in the homozygous state) [33]. Our previously reported experiments had demonstrated that substitution of E^441^ with A (Ala) reduced, but did not eliminate versican cleavage by ADAMTS5 in vitro and had revealed a cryptic ADAMTS5 cleavage site at E^438^-A^439^ (variant V1 sequence enumeration) [34]. Replacement of both glutamic acid residues with alanine, i.e., E^438^ to A + E^441^ to A eliminated ADAMTS5 processing in this region of the versican core protein [34]. With this preliminary evidence of specific point mutations that could abrogate versican processing, we used homologous recombination in mouse embryonic stem cells to replace E^438^ and E^441^ with A and generate transgenic mice with cleavage-resistant versican, an allele designated as *Vcan*^AA^. Here, we used this new allele to rigorously test the biological relevance of versican proteolysis at these two sites in the context of web regression and along with analysis of web regression in *Vcan* exon 7- or exon 8-specific mutants, we provide new insights on the role of ADAMTS proteases and versican in this process.

## Results

**Fig. 1:**
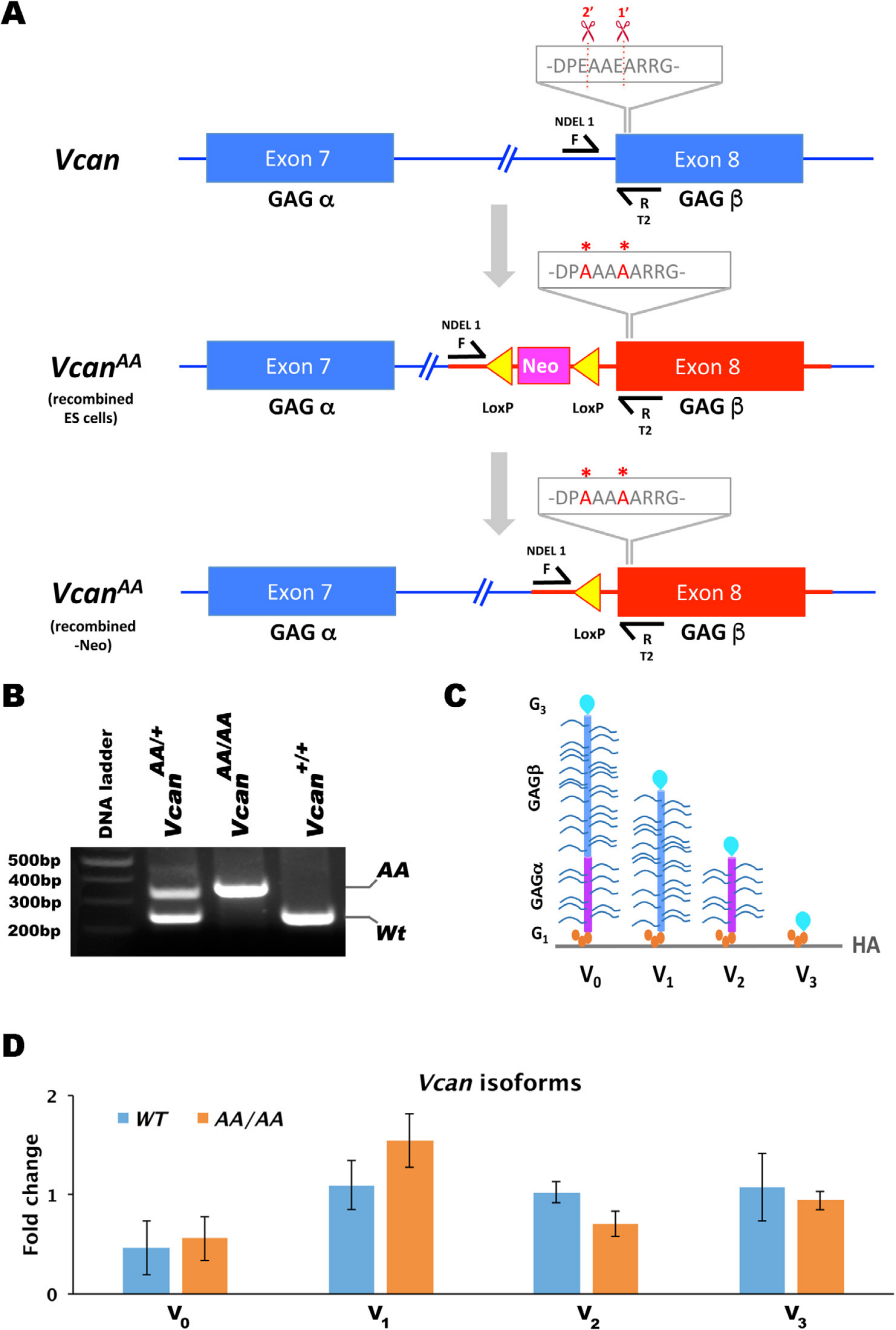
**Characterization of a new transgenic allele with cleavage-resistant versican.** (**A**) Transgenic strategy including genotyping strategy, showing *Vcan* exons 7 and 8 (with their encoded domains) (top), the engineered targeting vector (center) and the recombined allele after homologous recombination (bottom). The scissors indicate two previously identified cleavage sites in the GAGβ domain (sequence shown in single amino acid nomenclature, 1́ and 2́ indicate primary and secondary sites), and the asterisks indicate the introduced Ala replacement of Glu at these sites. The engineered exon 8 is shown in red with the relevant amino acid changes above. Neo indicates the neomycin-resistance cassette used for selection of transfected mouse embryonic stem cell (ESC) clones. Genotyping primers NDEL1F and RT2 are shown. Yellow triangles indicate LoxP sites used to delete the Neo cassette by homologous recombination after identification of correctly targeted embryonic stem cells. (**B**) Agarose gel electrophoresis of the PCR products obtained from genotyping with primers indicated in **A** showing the wild type and mutant (AA) bands and their detection in Wt, *Vcan*^AA/+^ and *Vcan^AA/AA^* mice. (**C**) Cartoon depicting the major versican isoforms V0-V3 (not drawn to scale). HA, hyaluronan. G1 and G3 indicate the versican globular domains. (**D**) The introduced mutations did not affect *Vcan* transcript levels, shown by RT-qPCR using isoform-specific primers. The arbitrary units are fold change relative to the control (wild type) group. (N = 4 mice from each genotype, error bars = S.E.M.). (For interpretation of the references to color in this figure legend, the reader is referred to the web version of this article.)

**Fig. 2:**
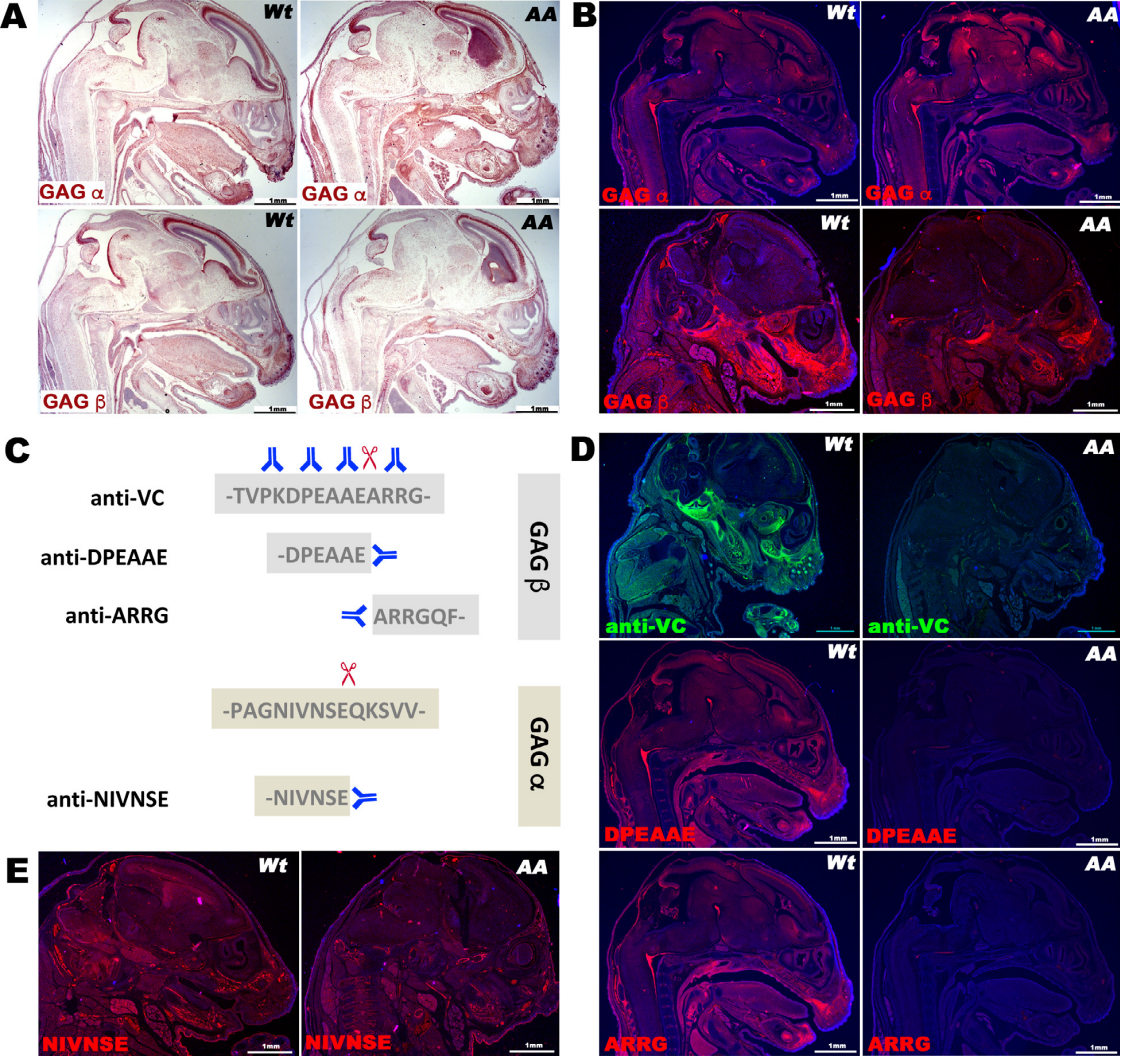
**Altered versican immunostaining and proteolysis resulting from the introduced mutations**. (**A-B**) The mutations did not affect distribution of versican transcripts shown in the craniofacial region by RNA in situ hybridization with GAGα (exon 7) or GAGβ (exon 8)-specific RNAscope probes (**A**, red signal) or protein distribution, shown by immunostaining with domain-specific antibodies (**B**, red signal), both in E14.5 embryos. (**C**) Epitopes of cleavage-relevant versican antibodies used in the present work. Scissors indicate the ADAMTS cleavage site. (**D**) The versican antibodies anti-VC (whose peptide epitope is centered on the cleavage site, unlike anti-versican GAGβ, which is generated to a longer spanning polypeptide) and anti-DPEAAE showed staining in sections from wild-type E14.5 embryos (Wt), but not *Vcan^AA/AA^* embryos, since the introduced mutations alter the epitopes of these antibodies. In contrast, anti-ARRGQF, which reacts with an N-terminal epitope exposed by cleavage at E^441^-A^442^ reacted similarly to anti-DPEAAE in Wt embryos, but also gave no staining in *Vcan^AA/AA^* embryos, demonstrating lack of cleavage at this site. In contrast, GAGα staining detected by anti-NIVNSE was present in both Wt and *Vcan^AA/AA^* embryos, since the E^405^-A^406^ cleavage site was unmodified. Scale bars in **A-E** = 1 mm. (For interpretation of the references to color in this figure legend, the reader is referred to the web version of this article.)

Homologous recombination of a targeting construct bearing the E^438^ + E^441^ mutations in ES cells successfully introduced the mutations into the *Vcan* locus (Fig. 1A, B). Both hemizygous and homozygous mutant mice were viable and fertile. qRT-PCR demonstrated that transcription of the four major *Vcan* splice isoforms was unaffected (Fig. 1C, D). Furthermore, RNA in situ hybridization demonstrated comparable distribution and intensity of *Vcan* mRNA using specific exon 7 and exon 8 probes, with each probe showing overlapping expression in wild-type and *Vcan*^AA/AA^ mutant embryos (Fig. 2A). Survival of *Vcan*^AA/AA^ mice indicated absence of the cardiac, vascular and hematopoietic developmental defects seen in *Vcan*^hdf/hdf^ mutants [11], [35], [36]. Thus, the specific core protein mutations that were introduced did not interfere with these essential functions of versican. Immunostaining using anti-GAGα antibody suggested comparable staining in wild-type and *Vcan*^AA/AA^ embryos (Fig. 2B). Weaker GAGβ immunostaining (with a commercial anti-GAGβ antibody) was seen in *Vcan*^AA/AA^ embryos, since the mutagenized Glu residues occurred within the immunogenic sequence of this antibody (Fig. 2B). Another GAGβ antibody, anti-VC [34], and anti-DPEAAE were unreactive in the mutant mice, which was expected, since the mutated sequence DPAAAA alters epitopes of each antibody (Fig. 2C,D) [34]. Specifically, since the peptide epitope of anti-VC, V^436^PKDP**EA**A**EA**RRGQ^445^ (human versican V1 isoform sequence enumeration; the residues forming the ADAMTS scissile bonds are in bold) is centered on the cleavage sites [34] it was expected to be non-reactive in *Vcan*^AA/AA^ mice, unlike the commercial versican antibody, anti-GAGβ, which is generated to a much longer polypeptide containing the anti-VC epitope and remains weakly reactive (Fig. 2B,D). We therefore used a neo-epitope antibody that detects the new N-terminus, ^442^ARRGQV, formed by cleavage at the GAGβ site, since this sequence was unaltered by targeted mutagenesis (Fig. 2C). In sections from *Vcan*^AA/AA^ mice, anti-ARRGQV reactivity was also lost, indicating that the engineered mutations abrogated cleavage as intended (Fig. 2D). To investigate cleavage at an ADAMTS processing site previously described in the human versican GAGα domain (NIVSFE^405^) [16] but uncharacterized in mice, we generated a new neo-epitope antibody, anti-NIVNSE, against the corresponding mouse sequence. The antibody reacted with the immunogen peptide with C-terminal residue E^405^, which was shown to be crucial for immunoreactivity, but not when E^405^ was succeeded by another residue (i.e., equivalent to the intact, uncleaved peptide sequence) (Supplemental Fig. 1). Anti-NIVNSE staining was present in both wild-type and mutant embryos (Fig. 2E). To explore whether abrogation of the processing sites would replicate ADAMTS-deficient phenotypes, *Vcan*^AA/+^ mice were intercrossed at the 3rd (N3) and 9th (N9) generation of outcrosses into the C57BL/6 strain, with the majority of studies being undertaken at N3. Variably penetrant white spotting, a phenotype seen in *Adamts20* and *Adamts9* mutants [20], [23], [27], [29], was initially seen in a small number of *Vcan*^AA/+^ and *Vcan*^AA/AA^ mice, but disappeared upon further outcrossing to the C57BL/6 strain. In regard to other ADAMTS protease mutant phenotypes, *Vcan*^AA/AA^ mice lacked cleft palate, a phenotype associated with reduced versican proteolysis in the palatal shelves of mice with combined reduction/inactivation of *Adamts9* and *Adamts20* [23], [26], [27], [28]. Versican cleavage at the E^441^-A^442^ site occurs during ovarian follicular maturation and ovulation, and was previously attributed to ADAMTS1, since *Adamts1* mutant mice had impairment of both processes [37], [38], [39]. *Vcan*^AA/AA^ female mice were fertile, suggesting no impact on ovulation and delivered pups without experiencing dystocia, a phenotype reported in mice with smooth muscle-specific conditional inactivation of *Adamts9* that was also accompanied by versican accumulation [32].

**Fig. 3:**
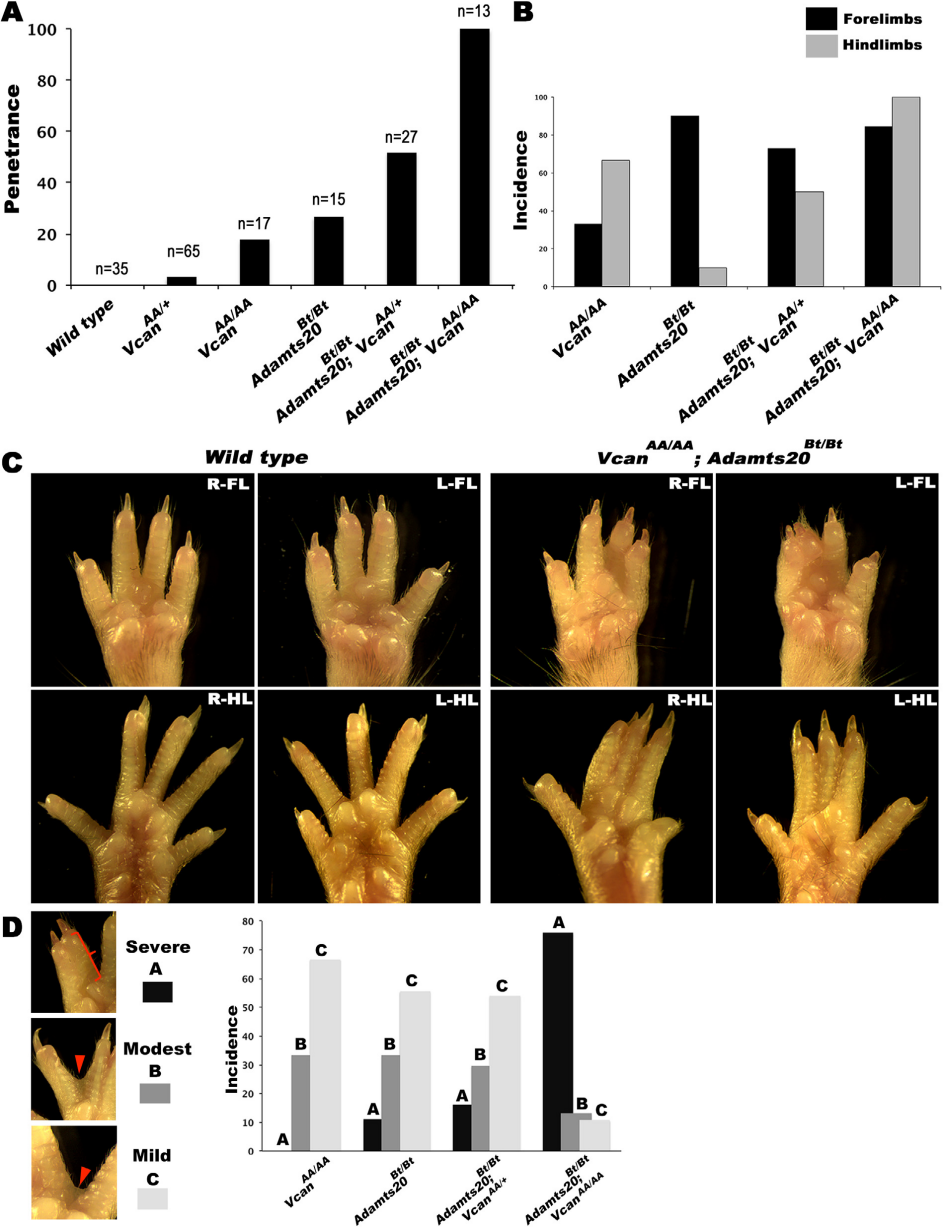
**More severe soft-tissue syndactyly (STS) in mice with cleavage-resistant versican with concurrent *Adamts20* deletion.** (**A**) Histogram showing incompletely penetrant STS in *Vcan^AA/+^* mice, higher penetrance in *Vcan^AA/AA^* mice and *Adamts20*^Bt/Bt^ mice, and complete penetrance in mice carrying both *Vcan^AA^* and *Adamts20* alleles. (**B**) *Vcan^AA/AA^* mice had a higher incidence of hindlimb STS, whereas *Adamts20*^Bt/Bt^ mice had a higher incidence of forelimb STS. *Vcan^AA/AA^*; *Adamts20*^bt/bt^ mice had near-complete involvement of both forelimbs and hindlimbs. (**C**) Illustration of STS severity in forelimbs and hindlimbs. The most severe involvement is observed in *Vcan^AA/AA^*; *Adamts20*^Bt/Bt^ mice*.* Their syndactylous limbs showed consistent involvement of the webs between hindlimb digits 2–3 and 3–4. (**D**) Classification system for the grade of STS and application to the indicated genotypes. The most severe grade was observed in *Vcan^AA/AA^*; *Adamts20*^Bt/Bt^ mice*.*

We undertook detailed analysis of interdigital web regression in *Vcan*^AA/AA^ mice, since this is the best characterized of the various defects associated with reduced versican processing. An observed low penetrance of hindlimb STS in *Vcan*^AA/+^ mice was increased in *Vcan*^AA/AA^ mice, but was nevertheless lower than that seen in *Adamts20*^Bt/Bt^ mice (Fig. 3A). Introducing one *Vcan*^AA/+^ allele in *Adamts20*^Bt/Bt^ mice doubled the penetrance, and STS was fully penetrant in *Vcan*^AA/AA^;*Adamts20*^Bt/Bt^ mice, affecting the webs between digits 2 and 3 and digits 3 and 4 (Fig. 3A). Forelimb STS was prevalent in *Adamts20*^Bt/Bt^ mice, as previously shown [4], whereas hindlimb STS was twice as common as forelimb STS in *Vcan*^AA/AA^ mice (Fig. 3B). Interestingly, *Vcan*^AA/AA^,*Adamts20*^Bt/Bt^ mice had roughly equivalent and near-complete penetrance of forelimb and hindlimb STS (Fig. 3B). The grade of STS, i.e., the extent of the syndactylous web along the length of the digit also progressed correspondingly in association with these genotypes (Fig. 3C, D).

**Fig. 4:**
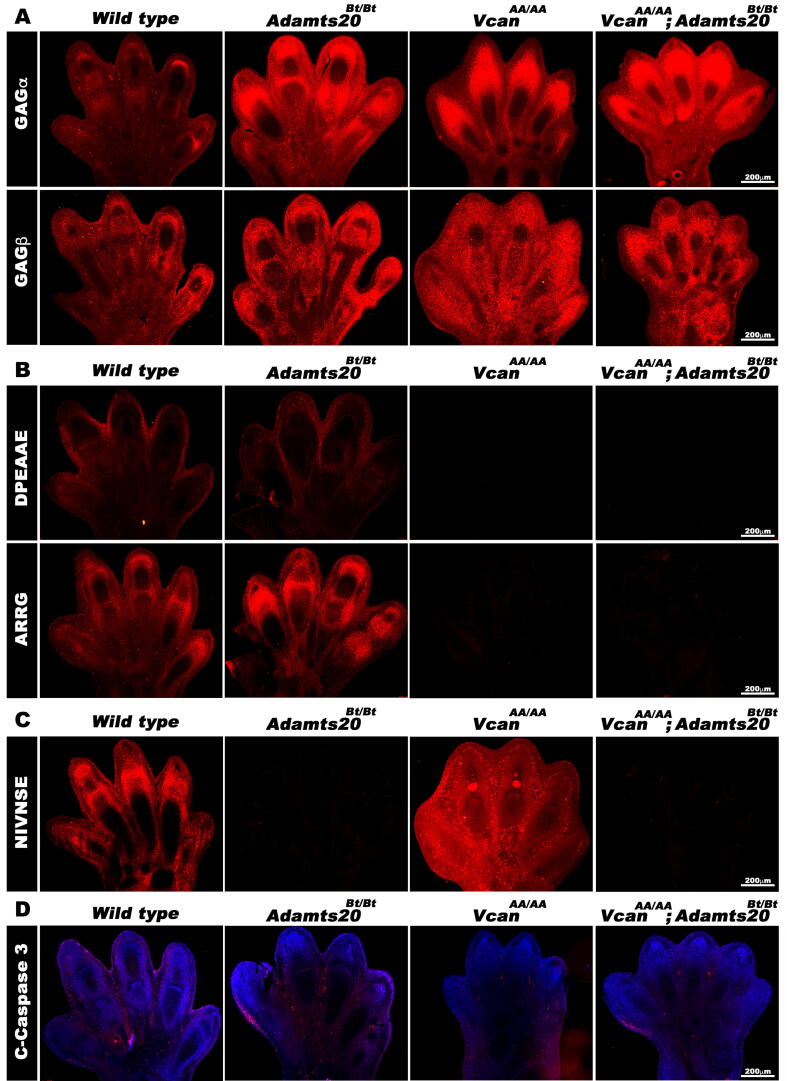
**Distribution of versican isoforms (A), cleaved versican (B,C) and apoptotic cells (D) in hind limbs of E13.5 mice of the indicated genotypes.** Note increased GAGα and GAGβ staining in each mutant allele shown, whereas anti-DPEAAE, as expected, gives no staining in the presence of mutant versican. Anti-ARRG staining is present in wild-type mice but also in *Adamts20*^Bt/Bt^ mice, consistent with expression of other ADAMTS proteases that can compensate for the absence of ADAMTS20, contributing to cleavage, but is absent in mice homozygous for the *Vcan* mutation. Anti-NIVNSE staining is absent in the *Adamts20*^Bt/Bt^ autopod, but evident in the *Vcan* mutant, where the GAGα processing site is intact. Reduced cleaved caspase staining is evident in the presence of mutant versican. Scale bars in **A-D** = 200 μm.

To ascertain the impact of the mutations on versican turnover, we stained for versican and its cleavage products in wild-type, *Vcan*^AA/AA^, *Adamts20*^Bt/^^Bt^ and *Vcan*^AA/AA^;*Adamts20*^Bt/Bt^ hindlimb autopods at 13.5 days of gestation. At this developmental stage, web regression is ongoing and incomplete so that it is not possible to anticipate which of the mutant webs will eventually fail to regress. Nevertheless, staining with both GAGα and GAGβ antibodies appeared to be stronger in each of the mutant autopods compared to wild-type autopods (Fig. 4A). As expected, we saw no anti-DPEAAE staining in any mutant with *Vcan*^AA/AA^ alleles (Fig. 4B). DPEAAE staining was detectable in the *Adamts20*^Bt/Bt^ webs (Fig. 4B), consistent with previous work showing that multiple ADAMTS proteases mediate versican proteolysis at the E^441^-A^442^ site [4]. Anti-ARRGQR gave no staining in interdigits from embryos having the *Vcan*^AA/AA^ genotype, demonstrating abrogation of cleavage after mutagenesis of Glu^338^ and Glu^441^ (Fig. 4B). Because we observed more intense GAGα staining in *Adamts20*^Bt/Bt^ mice (Fig. 4A) suggesting that ADAMTS20 might participate in versican cleavage in this core protein domain, we stained autopods of the various genotypes with anti-NIVNSE and found greatly reduced staining in *Adamts20*^Bt/Bt^ autopods (Fig. 4C). Finally, consistent with the prior finding of reduced apoptosis in syndactylous autopods from ADAMTS mutants, we observed reduced immunostaining for cleaved caspase-3, an apoptosis marker, in *Vcan*^AA/AA^, *Adamts20*^Bt/Bt^ and *Vcan*^AA/AA^*;Adamts20*^Bt/Bt^ autopods (Fig. 4D).

**Fig. 5:**
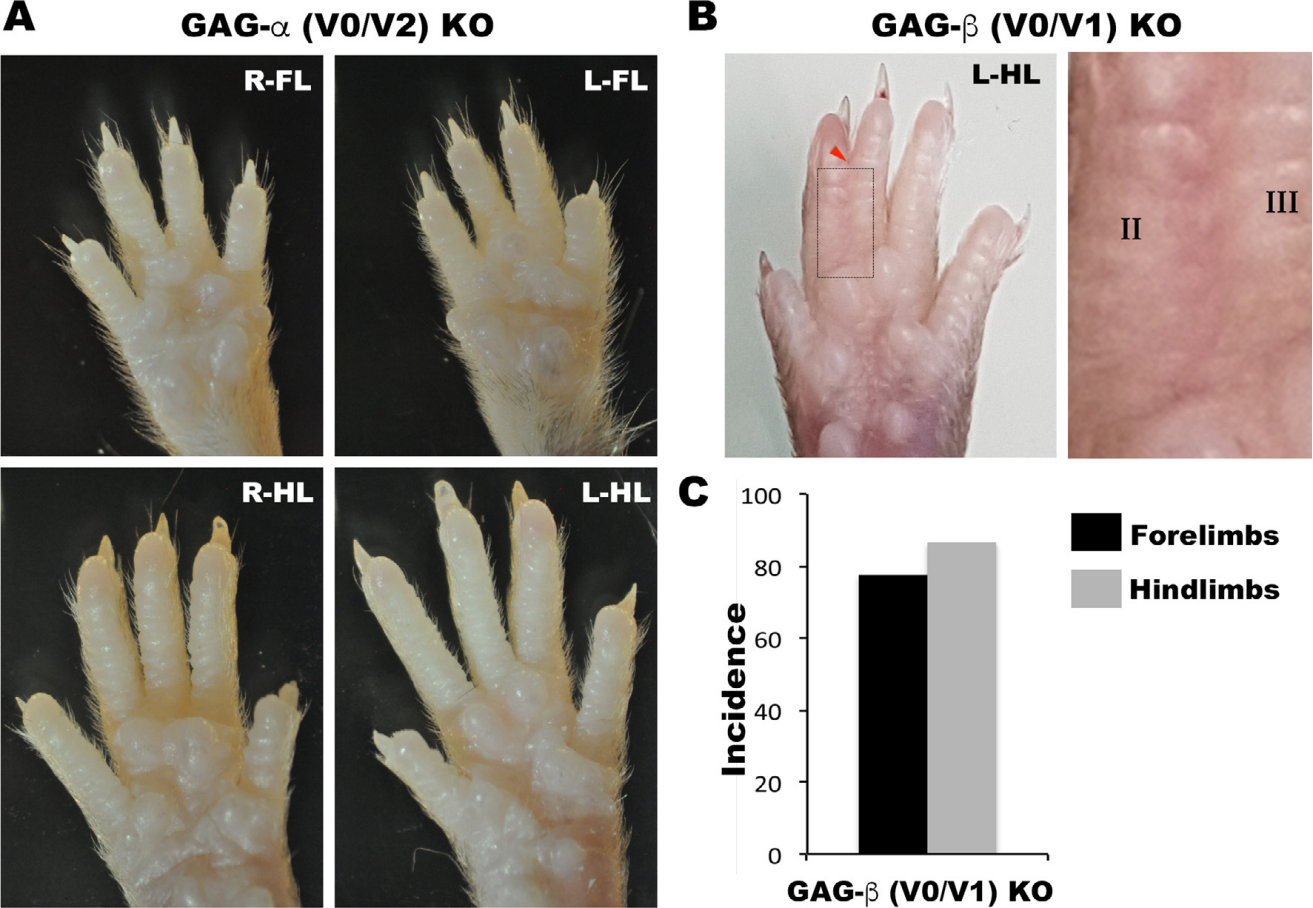
**Soft tissue syndactyly (STS) in *Vcan* GAG**β but not **GAG**α**knockout mice.** (**A**) STS is not seen in any of the limbs of the *Vcan* exon 7-lacking mice (GAGα, V0/V2 knockout). (**B-C**) STS was present with 100% penetrance in *Vcan* exon 8 mutant mice (GAGβ, V0/V1 knockout), affecting both forelimbs and hindlimbs, and typically the web between digits 2–3 and/or 3–4 (red arrowhead = STS, boxed area in **B** shown at higher magnification). (For interpretation of the references to color in this figure legend, the reader is referred to the web version of this article.)

Because prior work had demonstrated that versikine induced cell death in ADAMTS-deficient webs [4], we genetically interrogated the role of versican itself in interdigital web regression by analysis of the limbs of mutants specifically lacking either the GAGα domain [40] or GAGβ domain (Burin Des Roziers and Valleix, manuscript in preparation), which had not been previously done. In contrast to *Vcan*^hdf/hdf^ mutant mice [11], which lack all versican isoforms, the isoform-specific *Vcan* mutants survive past birth and into adulthood [40] [and Burin Des Roziers and Valleix, manuscript in preparation]. STS did not occur in the GAGα homozygous mutants which consistently showed fully separated digits, but was seen in all GAGβ homozygous mutants, specifically, 100% (98/98) of GAGβ domain homozygous mutant mice (Fig. 5 A, B). It affected forelimbs in 77% (76/98) of mice and hindlimbs in 86% (85/98) of mice, typically involving the 2–3 and 3–4 interdigit web (Fig. 5 B, C).

## Discussion:

In a prior in vitro analysis of versican cleavage by ADAMTS5, we demonstrated that mutation of the P1 and P3 glutamic acid residues (according to cleavage site nomenclature of Schechter and Berger [41]in the versican V1 core protein eliminated versican proteolysis at E^441^-A^442^ [34]. On the C-terminal side of the cleavage site, we found that elimination of two proximate GAG chains also reduced processing, likely via removing a putative binding site for the ADAMTS5 ancillary domain [34]. These findings suggested two alternatives for generating a mutant versican core protein that would be cleavage-resistant at the E^441^-A^442^ site. We chose to mutate E^438^ and E^441^ in the versican core protein in mice since mutation of the residues for GAG chain attachment and elimination of GAG chains could affect the intrinsic properties of versican. In contrast to our approach, Islam et al recently created a cleavage-resistant versican mouse mutant by leaving the DPEAAE sequence intact, but replacing the P1′ to P3′ residues (A^442^R^443^R^444^) by NVY [33]. Despite the intact DPEAAE sequence, *Vcan*(R/R) mice lacked anti-DPEAAE staining, demonstrating lack of cleavage [33]. Although anti-DPEAAE can no longer be used to detect versican cleavage in *Vcan*^AA/AA^ mutants, lack of anti-ARRGQF reactivity in *Vcan*^AA/AA^ embryos strongly supports elimination of cleavage at the E^441^-A^442^ site. Thus, each of these mouse mutants appears to have successfully abrogated embryo-wide proteolysis of versican at the E^441^-A^442^ site. Accordingly, hindlimb STS occurred in both *Vcan*^AA/AA^ and *Vcan*(R/R) mice [33], which carry different mutations engineered using different approaches, i.e., by homologous recombination in ES cells and CRISPR-Cas9 mutagenesis respectively. In contrast to *Vcan*(R/R) mice [33],*Vcan*^AA/AA^ mutants did not show lethality or other severe phenotypes. A detailed characterization of the *Vcan*^AA/AA^ mice suggested that the inserted mutations did not interfere with *Vcan* expression or isoform prevalence.

We focused on interdigital web regression during the embryonic period as a classic example of developmental tissue sculpting where versican proteolysis was previously suggested to have a crucial role [4]. The present findings provide novel insights on the role of versican and ADAMTS proteases in web regression. First, occurrence of partially penetrant STS in *Vcan*^AA/AA^ mutants suggests that versican processing at the E^441^-A^442^ site contributes to, but does not alone suffice for complete web regression. However, concurrent *Adamts20* deletion in *Vcan*^AA/AA^ mutants led to more highly penetrant STS. If ADAMTS20, previously shown to cleave versican at the E^441^-A^442^ site [20], cleaved it nowhere else, then its deletion should not have increased STS penetrance and severity. Therefore, we deduced that ADAMTS20 may cleave versican at one or more additional sites in the versican core protein or has one or more additional substrates in the ECM whose proteolysis is necessary for web regression. *Vcan*^AA/AA^ webs that were also lacking ADAMTS20 showed increased staining with a GAGα-specific antibody, indicating higher levels of V0/V2 versican, and we observed reduced anti-NIVNSE staining in these webs. Thus, web regression is associated with cleavage of versican at previously defined sites of ADAMTS proteolysis in both the GAGα and GAGβ domains. Whether ADAMTS20 cleaves versican at additional sites, or cleaves other ECM molecules to enable web regression remains unresolved for now and will be the subject of future studies.

The finding that GAGβ mutant webs but not GAGα mutant webs consistently have defective web regression is consistent with prior work identifying a pro-apoptotic role for versikine, a GAGβ-derived fragment, during web regression [4]. Furthermore, *Adamts20*^Bt/Bt^ mice haploinsufficient for a *Vcan* null allele (i.e., *Vcan*^hdf^) had more highly penetrant soft-tissue syndactyly than *Adamts20*^Bt/Bt^ mice, implying that a versican fragment was necessary for web regression [4]. Taken together, these findings strongly support an active role for versican in web regression, not merely as an ADAMTS substrate that is cleared during web regression, but as an essential contributor to this process as the source of versikine. Since the prior experiments that first suggested this possibility [4] used the *Vcan*^hdf^ mutant allele that lacked all isoforms, the present analysis now confirms that it is the GAGβ domain from which versikine is generated that is crucial. The homozygous GAGβ mutants however have a much higher penetrance of soft-tissue syndactyly than the Vcan^AA/AA^ mutants, suggesting that mutating a single cleavage site may not be sufficient to prevent versican proteolysis and that other cleavage sites may exist in the versican core protein that could generate versikine-like activity. This possibility is potentially supported by the higher penetrance resulting from combining the Vcan^AA/AA^ and *Adamts20*^Bt/Bt^ mutants. The data we present also suggests that although GAGα cleavage occurs during web regression, it is not causally involved in the process. However, it is not possible to precisely assess the relative contribution of versikine activity vs versican clearance during web regression. GAGβ proteolysis is undoubtedly essential, and perhaps even an obligate initiating event; however, which regions or motifs of versikine are critical and their underlying mechanisms are undefined. That the GAGα mutant did not develop syndactyly suggests that versikine activity may not reside in the G1 domain, which is common to both versikine and GHAP, but it could reside in the N-terminal segment of the GAGβ domain that constitutes the C-terminal region of versikine.

Mutagenesis of substrates to ascertain the significance of site-specific proteolytic cleavages has been previously used in only a few instances. For example, mice expressing collagen with an introduced mutation that abrogates the classic collagen I cleavage site (*Col1a1* (r/r) mice) provided vital information about the role of collagen turnover in bone, wound healing, atherosclerosis and post-partum uterine remodeling [42], [43], [44], [45], [46], [47], [48]. Mice with a mutation that rendered aggrecan resistant to the action of ADAMTS proteases at the E^373^- A^374^ site were protected from surgically induced osteoarthritis and inflammatory arthritis, indicating that ADAMTS proteases were major instruments of articular cartilage breakdown in arthritis [49]. Mice with cleavage-resistant reelin have demonstrated the importance of reelin processing by ADAMTS2 and ADAMTS3 in cerebral cortical and hippocampal development [50].

Genetic combination of cleavage-resistant substrate and protease mutants as reported here is rarely used, but can offer new insights. Conceptually, if a protease (e.g., ADAMTS20) has only one substrate (e.g., versican), which it cleaves at a single site (e.g., E^441^-A^442^), then genetically combining the cleavage-site mutant and protease mutant should not worsen the phenotype of the cleavage-site mutant. In the only other instance of a similar mutant inter-cross we are aware of, a combination of the *Col1a1* (r/r) mouse mutant and *Mmp2*^-/-^ mice led to more severe phenotypes than either transgene alone, highlighting collagen I cleavage at an alternative site by MMP2 [51]. In this regard, we are currently investigating other sites of ADAMTS proteolysis in the versican core protein.

In the future, *Vcan*^AA^ mutant mice can be investigated more thoroughly for additional morphogenetic roles, such as in the heart, craniofacial development and neural development, or in adult physiological and disease roles. *Vcan*(R/R) mice demonstrated accelerated wound healing attributed in part to greater myofibroblast activation [52], consistent with prior work showing that pericellular versican accumulation in dermal fibroblasts, achieved either by genetic inactivation of *Adamts5* or versican V1 overexpression led to enhanced TGFβ signaling and fibroblast-to-myofibroblast transition [18], [53]. Furthermore, combining the *Vcan*^AA^ mutant with other ADAMTS mouse mutants, such as was done here using *Adamts20*^Bt^, may well provide additional insights on the role of versican and ADAMTS processing of versican in the future.

## Methods

*Targeted mutagenesis of Vcan.* The Ensembl transcript *Vcan*-001 ENSMUST00000109546 (gene ENSMUSG00000021614) was used as the reference for design of a construct targeting the single *Vcan* locus on chromosome 13. Exon 8 of the 15 *Vcan* exons generating this transcript encodes the entire GAGβ domain. A ~ 9.2 kb region was subcloned from a C57BL/6 BAC clone (RP23-265 J12) into plasmid vector pSP72 (Promega, Madison, WI), containing an ampicillin selection cassette to construct the targeting vector. The point mutations were engineered by PCR mutagenesis. The engineered mutations in exon 8 altered the sequence from 5́GAAGCTGCAGAA3́ (translated as E^438^AAE^441^) to 5́GCAGCTGCAGCA3́ (translated as A^438^AAA^441^) and were located 269 nt downstream of the 5′ end of exon 8. The PCR fragment carrying the mutation was then inserted into the vector using a homologous recombination-based method. The targeting vector contained a 5′-homology arm of 3.0 kb juxtaposed to a FRT-LoxP-flanked self-excising neomycin resistance cassette (IV-UBS-FRT-LoxP flanked Neomycin cassette). This cassette was inserted 203 bp upstream of the intron 7-exon 8 splice site. The 3′ homology arm extended 5.7 kb downstream of the introduced mutations. The total size of the targeting construct (including pSP72 vector backbone, Neo cassette and a *Diphtheria* toxin cassette for negative selection) was 16.2 kb. The targeting vector construction was confirmed by restriction mapping and Sanger sequencing. 10 μg of the targeting vector linearized using *Not*I was electroporated into HF4 (129/SvEv × C57BL/6) (FLP hybrid) embryonic stem cells (ESC). After selection with G418, surviving ESC clones were expanded for PCR analysis to identify recombinant ESC. Neo was excised by FlpE recombinase during ESC clone expansion leaving behind a 118 nt FRT-loxP tandem site footprint. 4 positive clones identified as positive by Southern blot and PCR screening were selected for further expansion**.** For Southern blot, genomic DNA was digested with *Nhe*I, electrophoresed on a 0.8% gel and hybridized to a 398 bp probe external to the 5′ homology arm as shown. DNA from the HF4 strain was used as a wild-type control. Real-time quantitative PCR showed a single copy of the transgenic vector integrated in ES cells.

Targeted ESC from clone 151 were microinjected into C57BL/6 blastocysts. Resulting chimeras with a high percentage agouti coat color were mated to C57BL/6 wt mice to generate germline Neo deleted mice. 4 heterozygous mice (3 female,1 male) from one high percentage chimera derived from clone 151 were intercrossed to determine the effect of the transgene. Deletion of Neo and FlpE and the presence of the inserted mutations in these mice were confirmed by Sanger sequencing of PCR-amplified genomic DNA using appropriate primers. Outbreeding to the C57Bl/6 strain was undertaken for a further nine generations and mice were analyzed after the third and ninth intercross for the impact of the transgene on interdigital web regression. Mice were carefully examined for the incidence, extent and severity of STS as previously described [4]. Penetrance of STS was defined as a persistent web of any severity in at least one interdigit of a mouse [4].

*Other mouse transgenes*: The splice variant-specific versican GAGα mutant mouse strain Vcan^(tm1Zim)^ carries a translational stop codon preceded by an ER-retention signal in the GAGα-encoding exon 7. In consequence, versican isoforms V0 and V2 are absent in homozygous animals, while the isoforms V1 and V3 are normally expressed [40]. Mice lacking the GAGβ domain were generated by deleting the entire *Vcan* exon 8 by CRISPR-Cas 9 gene editing and will be described elsewhere (Burin Des Roziers, C. and Valleix,S., manuscript in preparation). qPCR analysis of the different *Vcan* transcript isoforms coupled with RNASeq analysis, and immunohistochemistry analysis of GAGβ homozygous mutant tissue has shown an absence of the V0/V1 transcript isoforms, a marked increase in V2/V3 transcript isoforms and absence of GAGβ immunostaining (Burin Des Roziers, C. and Valleix,S., manuscript in preparation).

*Antibodies, ELISA and Western blotting*. The generation of anti-^442^ARRGQF, to the new N-terminus generated by cleavage at E^441^-A^442^ was previously described [54]. In accordance with well-characterized methods for generation of neo-epitope antibodies [55], rabbits were immunized with the peptide immunogen CGGNIVNSE^405^ covalently coupled to keyhole limpet hemocyanin. Immune sera were purified by affinity chromatography using the immobilized peptide immunogen as the ligand and tested by ELISA against the same peptide. To determine the specificity of anti-NIVNSE for the neoepitope but not the bridging (intact) peptide sequence and for determining the critical requirements for antibody binding, we utilized ELISA against the immunogen peptides and a number of variant peptides. Peptides (4 μg/ml) were adsorbed to Nunc MaxiSorb plates and reacted with increasing dilutions of the respective antibodies. Anti-DPEAAE^441^, anti-GAGα and anti-VC were previously described [16], [17], [34].

*RNA In situ hybridization and immunofluorescence*: Mouse embryos were obtained at 13.5 and 14.5 days of gestation (E13.5 and E14.5) and fixed in 4% paraformaldehyde prior to paraffin-embedding. 7 μm thick sections were used for RNA in situ hybridization utilizing RNAscope technology (Advanced Cell Diagnostics) with specific *Vcan* probes for exon 7 and exon 8 [10], [28] as previously described [56]. Sections were used for antibody staining by an indirect immunofluorescence method after antigen retrieval, essentially as previously described [10], [28] using anti-cleaved caspase-3 (Asp175), (1:100, Cell Signaling, 9661), versican anti-GAGβ (catalog no. AB1033, MilliporeSigma), anti-GAGα (catalog no. AB1032, MilliporeSigma), anti-VC [34], anti-^42^ARRGQF [54] as well as anti-NIVNSE, which is described above. DAPI or hematoxylin were used as the counterstain as appropriate. Sections were photographed on an Olympus BX51 upright microscope (Olympus, Center Valley, PA) using a Leica DFC7000T camera and Leica Application Suite v4.6 imaging software (both from Leica, Wetzlar, Germany).

*Quantitative real time RT-PCR (qRT-PCR) analysis:* RNA was extracted from 23 day old wild type and *Vcan*^AA^ homozygous mouse tissues using TRIzol reagent (ThermoFisher catalog no. 15596026). Lung mRNA was used for *Vcan* V0, V1 and V3 qRT-PCR analysis and brain mRNA for *Vcan* V2 qRT-PCR analysis. 2 μg of RNA from each sample was used for cDNA synthesis using the high-capacity cDNA synthesis kit (Applied Biosystems, catalog no. 4368814). qRT-PCR was carried out using a CFX96 Touch Real-Time PCR detection system (BIO-RAD Laboratories) with the Bullseye EvaGreen qPCR mix (MIDSCI, catalog no. BEQPCR-S). 18 s ribosomal mRNA levels were used for normalization and relative expression of genes was calculated using the ΔΔCt quantification method. An unpaired, two-tailed Student *t*-test was used to determine statistical significance. The primer pairs used were: V0 primers, forward 5′ TTCACAGAACGCCACCCTTGAGTCC 3′ and reverse 5′ CTAGCTTCTGCAGCTTCCGGGTCC 3′; V1 primers, forward 5′ GCAGCTTGGAGAAATGGCTTTGACC 3′ and reverse 5′ CTAGCTTCTGCAGCTTCCGGGTCC 3′; V2 primers forward 5′ TCCTGGAGAATCTGTAACACAGCACCC 3′ and reverse 5′ CTCGGTAGGATAACAGGTGCCTCCG 3′; V3 primers forward 5′ ACTTCAGGCAGCTTGGAGAAATGGC 3′ and reverse 5′ ACTGGTCTCCGCTGTATCCAGGTGC 3′; 18s primers, forward 5′ TTGACGGAAGGGCACCACCAG 3′ , reverse 5′ GCACCACCACCCACGGAATCG 3′.

## Declaration of Competing Interest

The authors declare that they have no known competing financial interests or personal relationships that could have appeared to influence the work reported in this paper.
