# Supplementary material for: A new mouse mutant with cleavage-resistant versican and isoform-specific versican mutants demonstrate that proteolysis at the Glu441-Ala442 peptide bond in the V1 isoform is essential for interdigital web regression
Source: Matrix Biol Plus. 2021 May 14;10:100064. doi: 10.1016/j.mbplus.2021.100064 (PMC8233476; doi:10.1016/j.mbplus.2021.100064)
Supplement: Supplementary data 1 [file mmc1.docx]

**A mouse mutant with cleavage-resistant versican demonstrates that proteolysis at the Glu^441^-Ala^442^ peptide bond is essential for interdigital web regression**

Sumeda Nandadasa, Cyril Burin des Roziers, Christopher Koch, Karin Tran-Lundmark, María T. Dours-Zimmermann, Dieter R. Zimmermann, Sophie Valleix, Suneel S. Apte

**Data Supplement**

Supplemental Figure

**
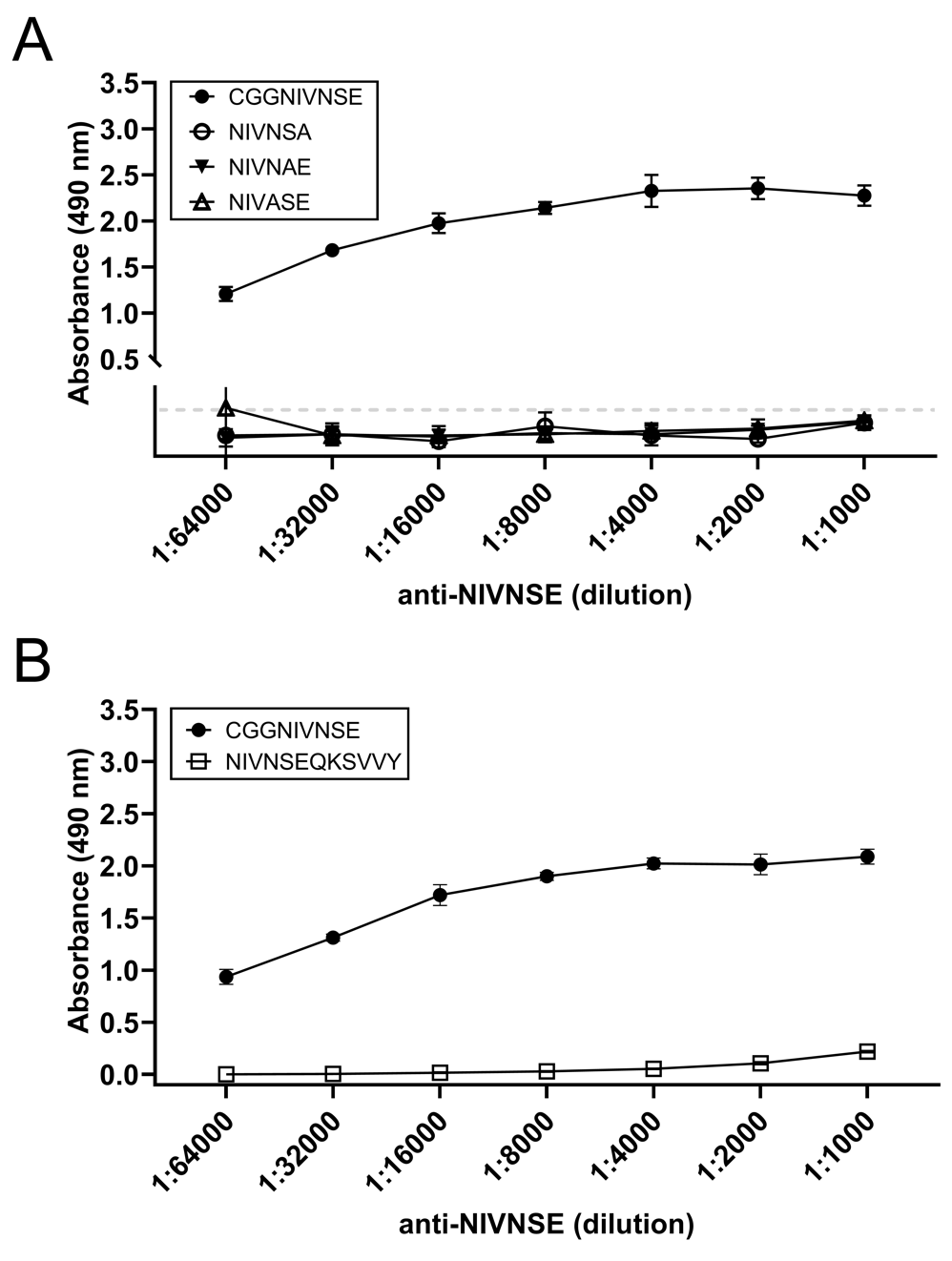
**

**Supplemental Figure.  The rabbit polyclonal anti-NIVNSE antibody is specific for the NIVNSE neoepitope.**  Anti-NIVNSE epitope specificity was determined using ELISA. Peptides representing the neoepitope, variant neoepitope peptides each with a single amino acid replaced by Ala as shown, or a peptide spanning the cleavage site (NIVNSEQKSVVY) were adsorbed to 96-well plates at a concentration of 4 µg/mL.  The reactivity of serially diluted anti-NIVNSE antibody, which was affinity purified using the CGGNIVNSE immunogen peptide, was then determined.  Purified anti-NIVNSE concentration was approximately 1 mg/mL before dilution.  **A**.  Replacement of each of the three most C-terminal amino acids of the neoepitope peptide with Ala disrupts anti-NIVNSE epitope recognition.  Each data point represents the mean of three replicate ELISAs with bars indicating the standard deviation.   Values are presented prior to background subtraction to illustrate that the absorbance of each disrupted neoepitope peptide was at or below the mean background (dashed grey line, mean absorbance = 0.051).  **B**. Anti-NIVNSE has very poor reactivity to the spanning peptide NIVNSEQKSVVY, demonstrating good specificity to the NIVNSE neoepitope.  Each data point represents the mean of three replicates, after background subtraction. Error bars indicate standard deviation.
